# A Novel MIP Gene Mutation Analysis in a Chinese Family Affected with Congenital Progressive Punctate Cataract

**DOI:** 10.1371/journal.pone.0102733

**Published:** 2014-07-17

**Authors:** Xuchen Ding, Nan Zhou, Hui Lin, Jianjun Chen, Chunyuan Zhao, Guangkai Zhou, J. Fielding Hejtmancik, Yanhua Qi

**Affiliations:** 1 Department of Ophthalmology, The Second Affiliated Hospital of Harbin Medical University, Harbin, China; 2 Department of Ophthalmology, Shanghai Tenth People’s Hospital and Tongji Eye Institute, Tongji University School of Medicine, Shanghai, China; 3 Ophthalmic Genetics and Visual Function Branch, National Eye Institute, National Institutes of Health, Bethesda, Maryland, United States of America; University of Arkansas for Medical Sciences, United States of America

## Abstract

Congenital cataracts are one of the leading causes of visual impairment and blindness in children, and genetic factors play an important role in their development. This study aimed to identify the genetic defects associated with autosomal dominant congenital progressive punctate cataracts in a Chinese family and to explore the potential pathogenesis. Detailed family history and clinical data were recorded, and all the family members’ blood samples were collected for DNA extraction. Linkage analysis was performed by microsatellite markers that are associated with punctate cataracts, and logarithm (base 10) of odds (LOD) scores were calculated using the LINKAGE program. Positive two-point LOD scores were obtained at markers D12S1622 (Z_max_ = 2.71 at θ = 0.0), D12S1724 (Z_max_ = 2.71 at θ = 0.0), and D12S90 (Z_max_ = 2.71 at θ = 0.0), which flank the major intrinsic protein of lens fiber (*MIP*) gene on chromosomal region 12q13. Direct sequencing of the encoding region of the *MIP* gene revealed a novel mutation (G>D) in exon 4 at nucleotide 644, which caused a substitution of glycine to aspartic acid at codon 215 (p.G215D) for the MIP protein. The mutation cosegregated with all patients with congenital progressive punctate cataracts, but it was absent in the healthy members. Bioinformatics analysis predicted that the mutation affects the function of the MIP protein. The wild type (WT) and G215D mutant of *MIP* were transfected with green fluorescent protein (GFP) into Hela cells separately, and it was found that the G215D mutant was aberrantly located in the cytoplasm instead of in the plasma membrane. In summary, our study presented genetic and functional evidence linking the new *MIP* mutation of G215D to autosomal dominant congenital cataracts, which adds to the list of *MIP* mutations linked to congenital progressive punctate cataracts.

## Introduction

Congenital cataracts, one of the most common causes of visual impairment, are responsible for approximately 10–30% of blindness cases in children [1]. Genetic factors play an important role in the development of congenital cataracts, and hereditary congenital cataracts are inherited primarily in an autosomal dominant pattern. The identified genes involved in congenital cataracts include ten crystalline genes (αA-crystallin [*CRYAA*] [2], αB-crystallin [*CRYAB*] [3], βA1-crystallin [*CRYBA1*] [4], βA4-crystallin [*CRYBA4*] [5], βB1-crystallin [*CRYBB1*] [6], βB2-crystallin [*CRYBB2*] [7], βB3-crystallin [*CRYBB3*] [8], γC-crystallin [*CRYGC*] [9], γD-crystallin [*CRYGD*] [10], and γS-crystallin [*CRYGS*] [11]), two cytoskeletal protein genes (beaded filament structural protein 2, phakinin [*BFSP2*] [12] and beaded filament structural protein 1, filensin [*BFSP1*] [13]), three transcription factor genes (heat shock transcription factor 4 [*HSF4*] [14], Maf-like protein [*MAF*] [15], and paired-like homeodomain 3 [*PITX3*] [16]), glucosaminyl (N-acetyl) transferase 2 (*GCNT2*) [17], chromatin-modifying protein-4B (*CHMP4B*) [18], transmembrane protein 114 (*TMEM114*) [19], and four membrane transport protein genes (major intrinsic protein of lens fiber [*MIP*] [20], lens intrinsic membrane protein 2 gene [*LIM2*] [21], gap junction protein [alpha 8, *GJA8*] [22], and gap junction protein [alpha 3, *GJA3*] [23]). Congenital cataracts are characterized by high genetic heterogeneity and clinical heterogeneity. For example, a mutation in CRYBB2 and CRYGD can cause CCA; in addition, MIP (also known as AQP0), CRYBA1, and GJA3 are related to congenital cataracts with punctate opacities.

MIP is expressed in the ocular lens, contributes to over 50% of the total membrane proteins in the lens fiber cell, and plays a role in maintaining lens transparency [24]. Mutations in the *MIP* gene have been linked to hereditary cataracts in humans and knockout mouse models [25]–[27], which further highlight the important role of MIP in maintaining lens transparency. To date, 11 mutations of *MIP* have been shown to be associated with congenital cataracts (c.97C>T [p.R33C] [28], c.401A>G [p.E134G] [29], c.413C>G [p.T138R] [29], c.530A>G [p.Y177C] [30], c.559C>T [p.R187C] [31], c.698G>A [p.R233K] [32], c.2 T>C [p.Met1] [33], c.494G>A [p.G165D] [34], IVS-1G>A [p.V203fs] [35], c.638delG [p.G213VfsX46] [36], and c.337C>T [p.R113X] [25]).

**Figure 1:**
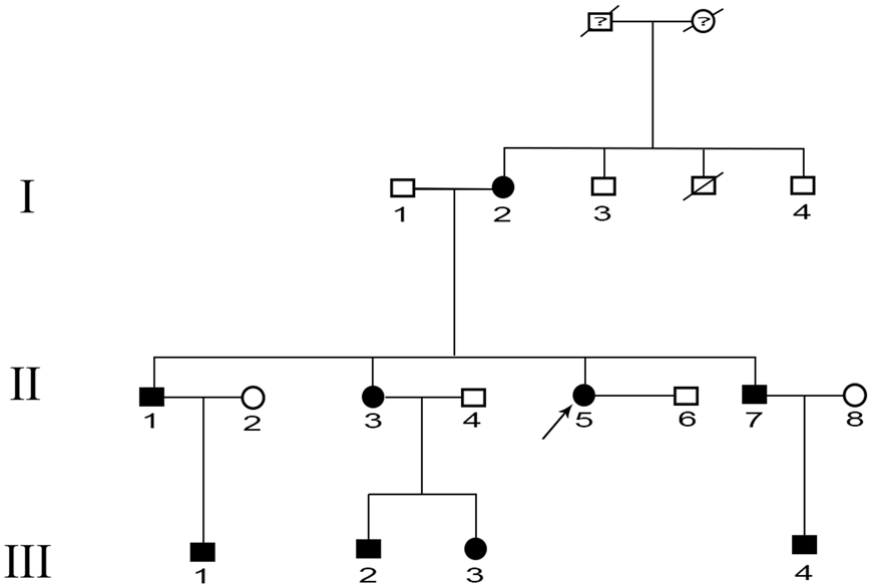
Pedigree of the family with autosomal dominant congenital progressive punctate cataracts. A three-generation pedigree with 16 members is shown. Squares and circles indicate males and females, respectively. Shaded shapes indicate affected individuals. The arrow indicates the proband.

**Figure 2:**
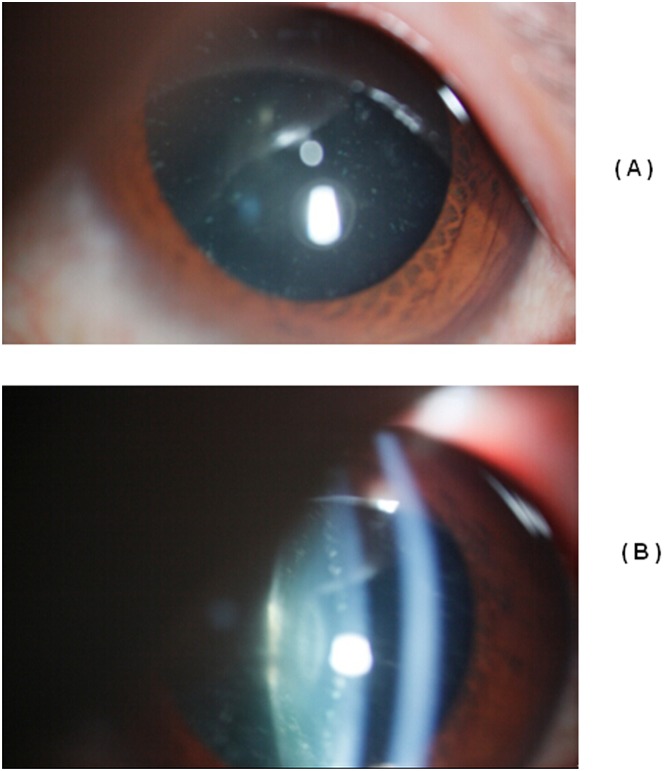
Slit-lamp photograph of the proband. Slit-lamp examination of the proband showed a cataract characterized as a punctate cataract.

In the present study, we identified that the p.G215D mutation of *MIP* was linked to congenital progressive punctate cataracts in a three-generation Chinese family (Figures 1 and 2). All family members with the G215D mutant of *MIP* have punctate cataracts, and the opacity degree of the lens increases with increasing age. Therefore, the aim of this study was to clone and express the G215D mutant of *MIP* and compare its properties with those of the normal or wild-type (WT) *MIP*.

## Methods

### Ethics Statement

After explaining the nature and possible consequences of the study, written informed consents were obtained from subjects including the guardians on behalf of the minors enrolled. All experiments were approved by the Institutional Review Board of Harbin Medical University (Harbin, China) and were conducted according to the principles in the Declaration of Helsinki.

### Patient ascertainment and genomic DNA preparation

A three-generation Chinese Han family with congenital progressive punctate cataracts from Shenzhen province was identified as shown in Figure 1. All members of the pedigree were given complete ophthalmologic examinations and did not have any other eye disease. One hundred healthy persons were recruited as the normal controls. After informed consent, 5 ml of venous blood from each subject was collected in a BD Vacutainer (BD, San Jose, CA) containing EDTA. Genomic DNA was extracted by QIAamp DNA Blood Mini Kits (QIAGEN Science, Germantown, MD).

### Genotyping and linkage analysis

Fluorescently labeled microsatellite markers, which have been reported to be associated with punctate cataracts, were used for the linkage analysis. Two-point linkage analysis was performed with MLINK from the LINKAGE program package. The disease locus was specified to be an autosomal dominant trait, with a disease allele frequency of 0.0001. The allele frequencies of the markers are considered to be equally distributed.

### Mutation screening

The coding exons and the flanking regions of the candidate gene were amplified by polymerase chain reaction (PCR) with the primers listed in Table 1. The sequencing was carried out with an ABI 3130XL Genetic Analyzer (Applied Biosystems, Foster City, CA), and the results were analyzed using Chromas (version 1.0) software.

**Table 1:** Primer sequences.

Exon	Forward	Reverse	Annealing temperature
MIP-1	TCTCGGCTCATCTCCCAGTT	GGCAATAGAGAGACAGGACAC	61.4°C
MIP-2	TGAAGGAGCACTGTTAGGAGATG	AGAGGGATAGGGCAGAGTTGATT	61.4°C
MIP-3	CCAGACAGGGCATCAGT	CCAGACAGGGCATCAGT	58°C
MIP-4	AAGGTGTGGGATAAAGGAGT	TTCTTCATCTAGGGGCTGGC	60°C

### Bioinformatics analysis

The WT and mutant MIP protein sequences were analyzed with the PolyPhen tool to predict whether the amino acid substitution affects the structure and function of the protein. The amino acid sequences of MIP from several different species were obtained from NCBI GenBank, and conservation analysis was performed by CLC Main Workbench Software (Aarhus, Denmark). The hydrophobic properties of the mutant and WT amino acid sequences were analyzed by Misc Protein Analysis software, respectively.

### Plasmid construction

The expression vector for G215D mutant of *MIP* was constructed by using site-directed mutagenesis with the following primers: sense primer 5′-GGAGGGGGTCTGGACAGCCTCCTGTAC-3′; antisense primer 5′-GTACAGGAGGCTGTCCAGACCCCCTCC-3′. Mutation was confirmed through DNA sequencing. pGFP-WT and pGFP-G215D resultant plasmids were constructed to create GFP-MIP fusion proteins, the open reading frames of WT and G215D mutant of *MIP* were amplified by PCR with the following primers: sense primer 5′-AGT ACC TCG AGA TGT GGG AAC TGC GAT CAG C-3′; antisense primer 5′-CTA CGA AGC TTC AGG GCC TGG GTG TTC AGT T-3′. Products were then cloned into Xho I and Hind III digested pGFP-N1 vector.

### Cell culture and transfection

Human cervical cancer (Hela) cells were maintaining in Dulbecco’s modification of Eagle’s medium (DMEM) supplemented with 10% fetal bovine serum (FBS), 100 mg/ml penicillin and 100 mg/ml streptomycin in a humidified atmosphere containing 5% CO_2_ at 37°C. Transfection was carried out using Lipofectamine 2000 (Invitrogen Corporation, Carlsbad, CA, USA).

### Expression and subcellular localization

Hela cells were plated in 6-well plates 24 h prior to transfection at approximately 60% confluency. According to the manufacturer’s protocols, GFP-MIP expression constructs containing WT and G215D mutant were transfected using Lipofectamine 2000, separately. The empty vector pGFP-N1 was transfected as a control. 48 hours after transfection, the cells were analyzed by fluorescence microscopy.

### Western blot analysis

After transfected with MIP-WT or MIP-G215D plasmids separately, HEK 293t cells were harvested and lysed in lysis buffer (50 mM Tris-HCl, 1% NP40, 150 mM NaCl, 1 mM EDTA and 1 mM PMSF) for 30 min at 4 uC. Total proteins were extracted and separated by 12% SDS/PAGE gels, and then transferred to PVDF membranes, separately. The membranes were incubated with antibodies of anti-β-actin (Cell Signaling Technology, Inc, USA) and anti-GFP (Abcam, Inc, Cambridge, MA). The signals were visualized by using the chemiluminescent substrate method with the SuperSignal West Pico kit. (Pierce Co, Rockford, USA).

## Results

**Figure 3:**
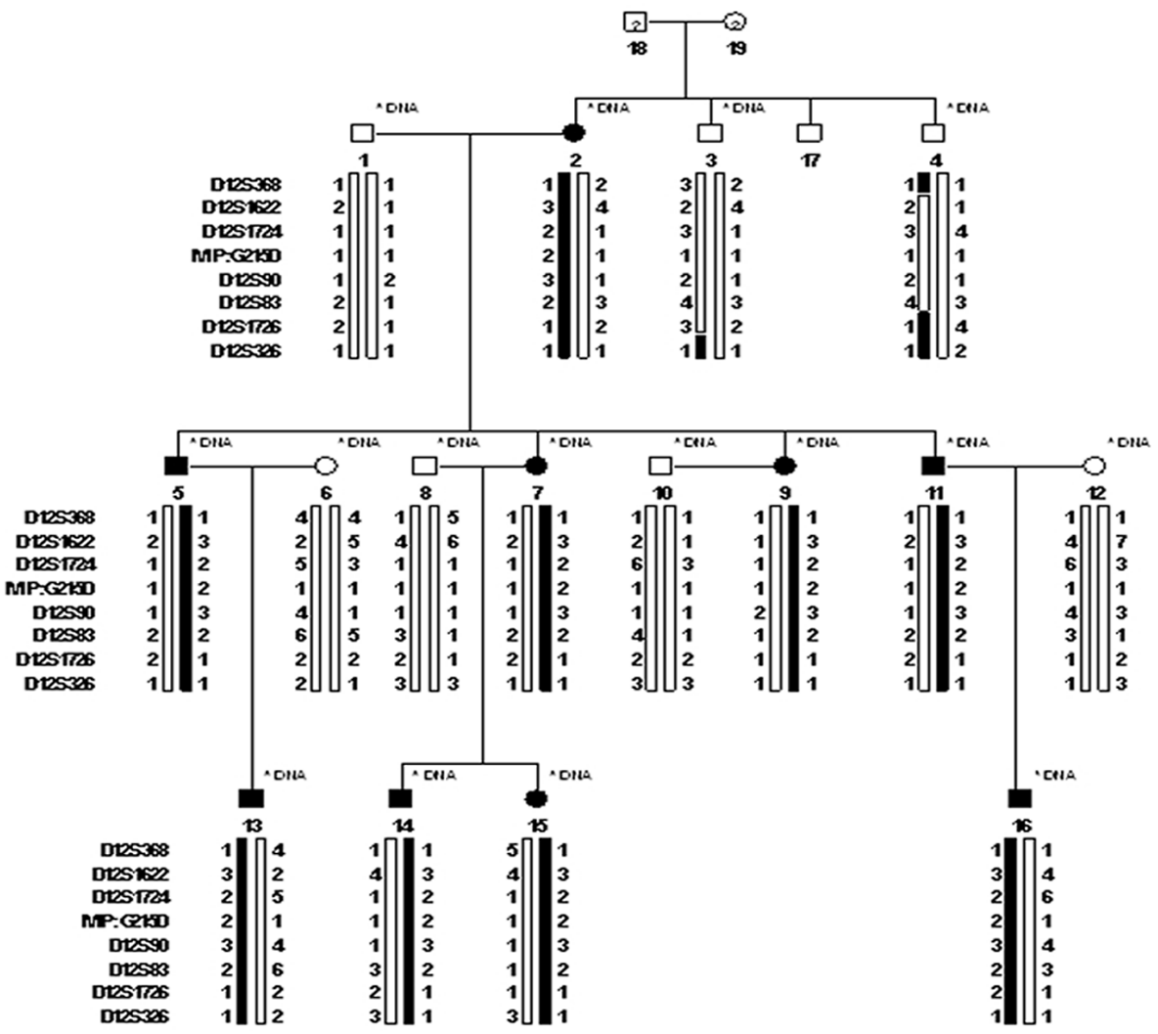
Haplotype of the family. Eight markers close to *MIP* were used. The disease haplotype (represented by the black bar) cosegregated with all affected family members from marker D12S1622 to D12S83 but was not shared with unaffected members.

Nine family members (I:2, II:1, II:3, II:5, II:7, III:1, III:2, III:3, and III:4) were diagnosed as having congenital progressive punctate cataracts, and they had no other ocular or systemic abnormalities (Figure 1). Progressive punctate cataracts were exhibited fully in these patients (Figure 2). Among them, three had undergone cataract surgery. By linkage and haplotype analysis, we obtained positive two-point logarithm (base 10) of odds (LOD) scores with markers at 12q13. Table 2 shows the linkage analysis results of markers D12S1622 (Z_max_ = 2.71 at θ = 0.0), D12S1724 (Z_max_ = 2.71 at θ = 0.0), and D12S90 (Z_max_ = 2.71 at θ = 0.0). The haplotype analysis showed complete cosegregation in all nine patients (Figure 3).

**Table 2:** Result of linkage analysis.

MARKER	cM	Mb	0	0.01	0.05	0.1	0.2	0.3	0.4	Z_max_	θ_max_
D12S368	66.03	52.63	−∞	−0.51	0.09	0.27	0.32	0.22	0.07	0.32	0.2
D12S1622	69.82	54.76	2.71	2.66	2.46	2.21	1.65	1.04	0.44	2.71	0
D12S1724	69.92	54.87	2.71	2.66	2.46	2.21	1.65	1.04	0.44	2.71	0
MIP:c.644G>A, p.G215D		56.84	2.71	2.66	2.46	2.21	1.65	1.04	0.45	2.71	0
D12S90	71.61	58.42	2.71	2.66	2.46	2.21	1.65	1.04	0.45	2.71	0
D12S83	75.17	60.88	1.51	1.47	1.34	1.18	0.83	0.46	0.13	1.51	0
D12S1726	75.77	62.45	−∞	−0.82	−0.22	−0.05	−0.01	−0.01	−0.01	−0.01	0.2
D12S326	86.4	77.97	−0.81	−0.72	−0.5	−0.33	−0.15	−0.06	−0.01	−0.01	0.4

**Figure 4:**
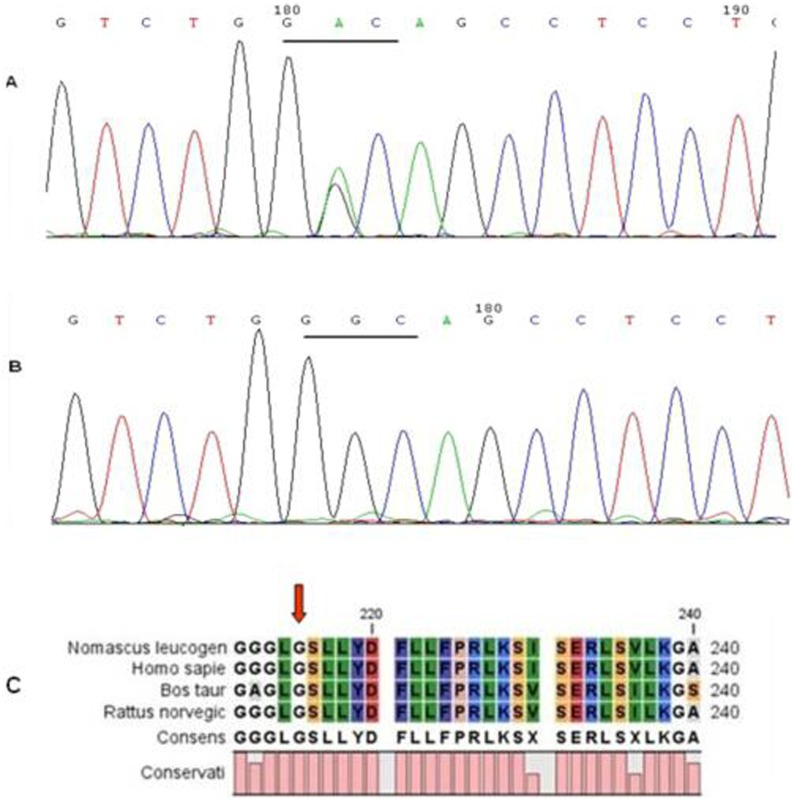
Mutation analysis of the *MIP* gene. (**A**) Partial nucleotide sequence of *MIP* from a patient of the family. The sequence in patients showed a heterozygous G>A conversion, resulting in a substitution of glycine to aspartic acid at codon 215. (**B**) The healthy members of the family and 100 control subjects lacked this nucleotide change. (**C**) Phylogenetic conservation analysis. The alignment of the *MIP* sequence with the corresponding segments of diverse species is shown. Glycine 215 was highly conserved in MIP proteins from several species.

Direct sequencing revealed a heterozygous change, G>A, at position 644 (c.644G>A) of the *MIP* gene, which leads to the substitution of a glycine in the WT to an aspartic acid at codon 215 (p.G215D). Glycine is highly conserved across various species, as determined from the NCBI database. This mutation cosegregated well with all affected individuals and was not found in unaffected family members or in the 100 unrelated normal controls (Figure 4).

**Figure 5:**
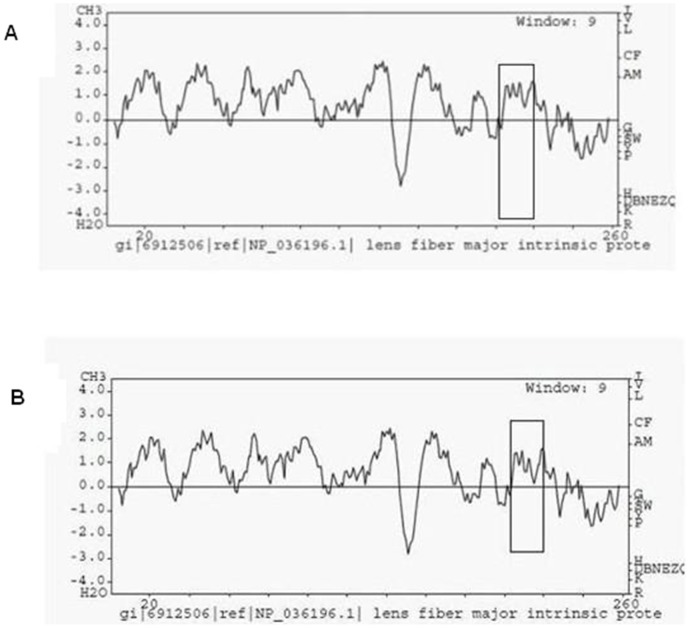
Hydrophobicity prediction of the WT (A) and the mutant (B) MIP protein. The substitution region on the MIP protein is boxed. It is obvious that the mutant has a lower hydrophobicity in this region compared with the WT.

PolyPhen analysis demonstrated that the substitution at position 215 of *MIP* from glycine to aspartic acid scored 0.811, which meant that this mutation is predicted to be “possibly damaging”. In comparison with the WT-MIP protein, it is obvious that the mutant has a lower hydrophobicity in this region (Figure 5).

**Figure 6:**
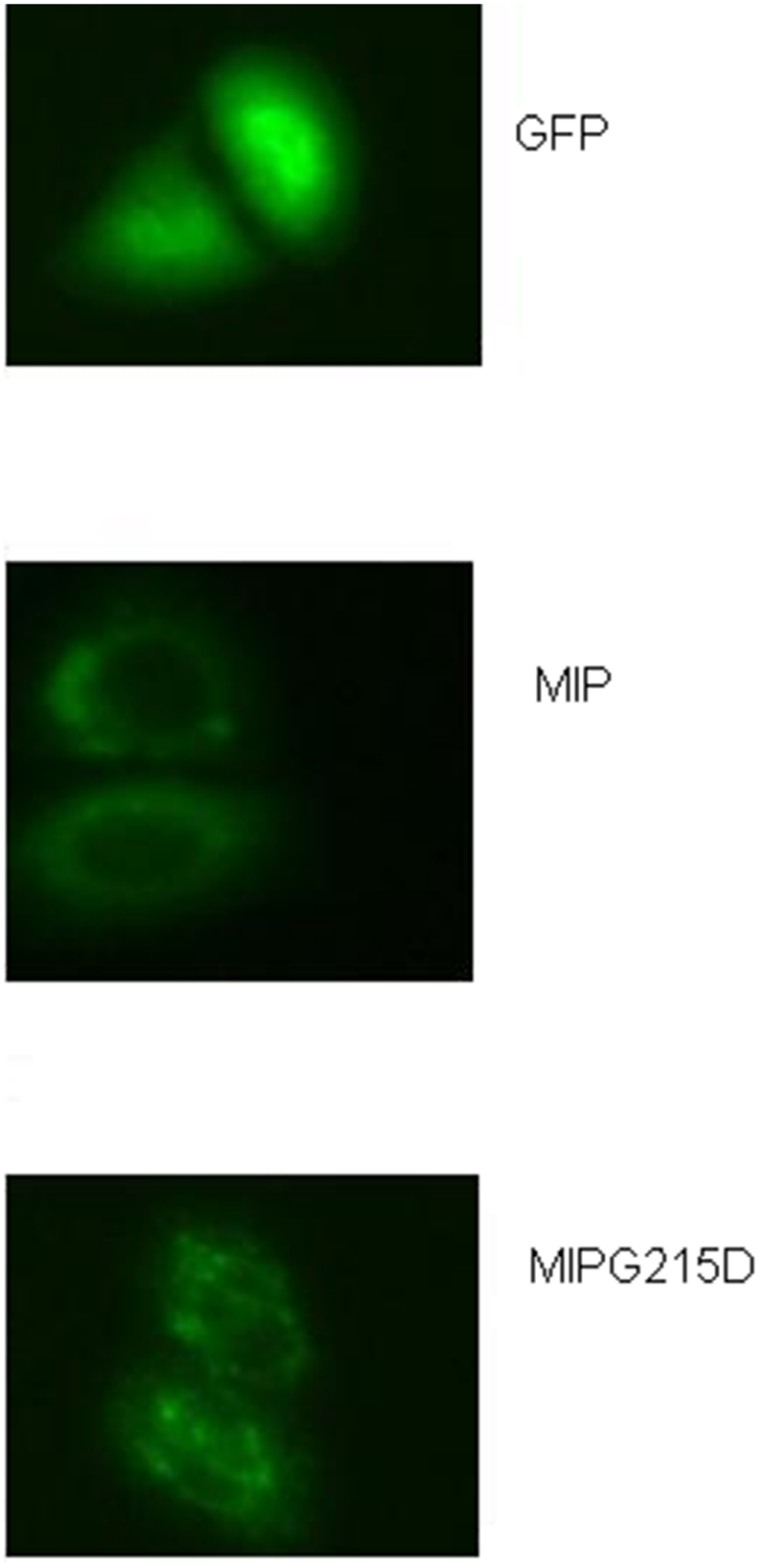
Subcellular localization of MIP. Localizations of WT and G215D-MIP proteins in transfected Hela cells were viewed by fluorescence microscopy. (**A**) The empty vector pGFP-n1 was located in both the nucleus and cytoplasm. (**B**) The expressed WT-MIP was located in the plasma membrane. (**C**) The G215D-MIP was aberrantly located in the cytoplasm.

We tested the subcellular location of WT-MIP and G215D-MIP after transient transfection of Hela cells by fluorescence microscopy. As shown in Figure 6, the empty vector pGFP-N1 was located in both the nucleus and cytoplasm, while the expressed WT-MIP was located in the plasma membrane. In contrast, G215D-MIP was aberrantly located in the cytoplasm, indicating that the G215D mutation prevented its localization in the plasma membrane, accurately.

**Figure 7:**
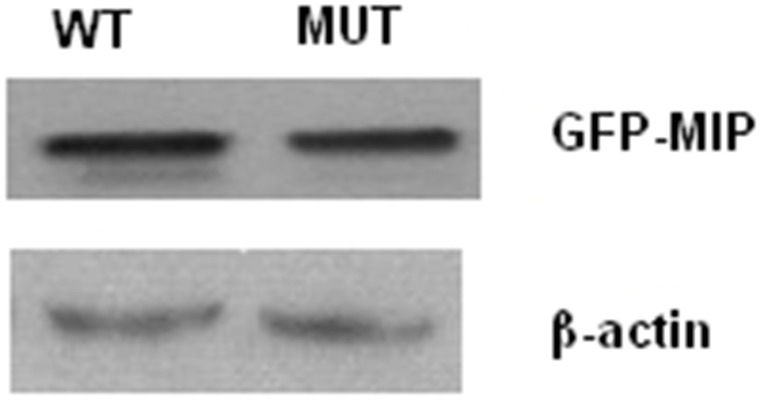
The protein levels of WT-MIP and G215D-MIP. Western blot analysis indicating that the mutation did not result in the expression or instability of the protein.

Western blot analysis revealed that WT-MIP and G215D-MIP had similar protein levels in western blot analysis of the cell lysates (Figure 7), indicating that mutation did not result in the expression or instability of the protein.

## Discussion

In the present study, a novel mutation of the MIP gene was identified in exon 4 at nucleotide 644, which caused a substitution of glycine to aspartic acid at codon 215 (p.G215D) in a Chinese family with congenital progressive punctate cataracts. The G215D MIP mutant was found to cosegregate with the disease phenotype in the family and was not present in unaffected family members or in 100 normal controls.

The *MIP* gene, also known as the MIP26 or AQP0 gene, encodes a 263-amino acid peptide. AQP0 is abundant and contributes to over 50% of total membrane fiber proteins [37]. As water pores confer rapid movements of water across plasma membranes, AQP0 is considered to be essential for the lens microcirculation system and the maintenance of lens transparency [38], [39]. It has been widely reported that mutations of *MIP* in humans and mice are involved in the induction of cataracts [40]–[43].

As aquaporin, MIP has a unique structure with six transmembrane bilayerspanning domains (H1–H6), three extracellular loops (A, C, and E), two intracellular loops (B and D), and the NH2- and COOH-terminal intracellular domains. To the best of our knowledge, this is the third mutation of the *MIP* gene in transmembrane bilayer-spanning domains (H6) and the first substitution mutation in H6 that can cause autosomal dominant congenital cataracts. The first mutation in H6 of MIP (C.638delG) was reported to be associated with polymorphic cataracts in 2006 [36], and the second mutation of MIP (IVS-1G>A) was found to be associated with nuclear “snail-like” cataracts in 2009 [35]. The functional consequences of the 638G deletion have been identified, showing that the mutant protein was located in the endoplasmic reticulum and induced cellular cytotoxicity [36]. Similarly, Prior studies of mutant MIP proteins have shown a loss of water permeability function and impaired trafficking in expression systems [43], [44]. Our findings were consistent with the results of Senthil Kumar et al [43], who identified the G165D MIP mutation and confirmed that this mutant was involved in congenital lamellar cataracts in a South Indian family. In addition, the expression of MIP and G165D-MIP in Xenopus oocytes and Hela cells suggested that substitution of the conserved glycine residue leads to improper trafficking of AQP0 and destruction of water channel function.

In summary, the current study presented genetic and functional evidence linking a new mutation of MIP (G215D) to autosomal dominant congenital cataracts. Our results showed that G215D-MIP had a lower hydrophobicity and was aberrantly located in the cytoplasm of Hela cells, while WT-MIP was mainly detected in the plasma membrane. We speculate that G215D-MIP would be unable to exert channel functions. Since AQP0 has several functions and plays an adhesive molecule role in the lens, it is likely that, besides channel functions, G215D-MIP would be unable to exert many other functions. Further studies, such as research on protein aggregation in solution and animal models, are needed in order to provide insights into the molecular consequence of this mutation.
